# Defects in Germinal Center Selection in SLE

**DOI:** 10.3389/fimmu.2015.00425

**Published:** 2015-08-14

**Authors:** Megan Woods, Yong-Rui Zou, Anne Davidson

**Affiliations:** ^1^Center for Autoimmunity and Musculoskeletal Diseases, Feinstein Institute for Medical Research, New York, NY, USA

**Keywords:** SLE, B cell, germinal center, autoimmunity, tolerance mechanisms

## Abstract

Germinal centers (GCs) are the primary site at which clonal expansion and affinity maturation of B cells occur. B cells encounter antigen and receive T cell help in the GC light zone (LZ) and then migrate to the dark zone where they proliferate and undergo somatic mutation before cycling back to the LZ for further rounds of selection. Tolerance to autoantigens is frequently lost *de novo* as GC B cells undergo class switching and somatic mutation. This loss of tolerance is regulated by a variety of mechanisms including cell death, failure to compete for T cell help, and failure to differentiate into effector cells. Systemic lupus erythematosus (SLE) is characterized by loss of tolerance to nucleic acid antigens. While defects in tolerance occur in the naïve repertoire of SLE patients, pathogenic autoantibodies also arise in the GC by somatic mutation from non-autoreactive precursors. Several B cell defects contribute to the loss of GC tolerance in SLE, including polymorphisms of genes encoded by the Sle1 locus, excess TLR7 signaling, defects in FcRIIB expression, or defects of B cell apoptosis. Extrinsic soluble factors, such as Type-1 IFN and B cell-activating factor, or an increased number of T follicular helper cells in the GC also alter B cell-negative selection. Finally, defects in clearance of apoptotic debris within the GC result in BCR-mediated internalization of nucleic acid containing material and stimulation of autoantibody production by endosomal TLR-driven mechanisms.

## Introduction – Germinal Center Formation and Structure

In quiescent lymphoid follicles, a chemokine gradient restricts CCR7^+^/PSGL-1^+^ T cells to a central area and CCR7^−^/CXCR5^+^ B cells to a ring surrounding the T cell zone. Major changes in this structure occur upon antigen encounter. Activated T cells downregulate CCR7 and PSGL-1 and upregulate CXCR5, promoting their migration to the interfollicular zone where they contact antigen-activated B cells that have upregulated CCR7. The interaction of ICOSL on B cells with ICOS on T cells initiates the differentiation of T follicular helper cells (TFH), whereas T cell help promotes the expansion of extrafollicular (EF) B cell foci (1–3). B cell fate decision is then regulated by several factors including the affinity of the B cell receptor (BCR) for antigen, costimulatory signals from T cells, and the cytokine milieu (4, 5). Some B cells will remain outside the follicle and upregulate Blimp1, becoming short-lived low-affinity plasmablasts. Other B cells downregulate EBV-induced molecule-2 (EBI2) that interacts with a chemokine-like gradient established by 7α,25-dihydroxycholesterol (6–9); these cells, as well as CXCR5^+^ TFH, move inside the follicle to form the germinal center (GC) (6). Expression of particular transcription and survival factors including Bcl6 and IRF4 in T cells and Bcl6, Myc, and Mcl1 in B cells is required for the differentiation and survival of GC cells [reviewed in Ref. (10, 11)].

**Figure 1:**
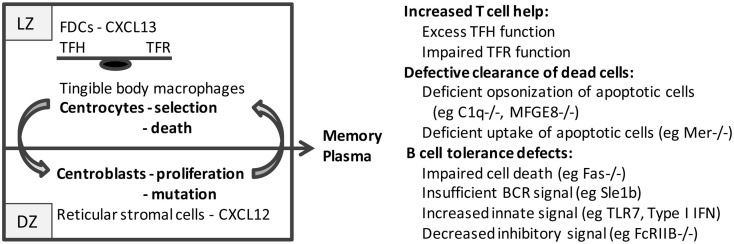
**Schematic of the germinal center: germinal centers consist of two major anatomic regions**. The major cell types in the light zone (LZ) and dark zone (DZ) are shown. B cell centrocytes undergo selection in the light zone and then migrate to the dark zone where the B cell centroblasts proliferate and undergo somatic mutation before cycling back to the light zone. After several cycles, B cells differentiate into memory cells or plasma cells and exit the germinal center. Defects in germinal center cells or functions that result in spontaneous germinal center formation and loss of tolerance to nucleic acid antigens are shown on the right.

As the GC matures, stromal cells orchestrate the spatial segregation of centrocytes and centroblasts into the light zone (LZ) and dark zone (DZ) regions, respectively (Figure 1). Follicular dendritic cells (FDC), usually located inside quiescent B cell follicles (12), form clusters within the developing LZ; these cells capture and retain immune complexes via the complement receptors, CD21 and CD35 (13, 14). FDCs also produce CXCL13 that recruits CXCR5^+^ centrocytes and TFH cells into the LZ, thus forming a platform for cognate B–T cell interactions (15). By contrast, CXCR4^hi^ centroblasts are segregated in the DZ by reticular cells expressing high levels of CXCL12 (16). Selection of B cells begins as early as the pre-GC stage and continues in the LZ where centrocytes are exposed to FDC-bound native antigen. High-affinity B cells endocytose and present more antigen to T cells thereby competing most efficiently for help from TFH. Centrocytes then upregulate CXCR4 and migrate into the DZ where they proliferate and accumulate somatic mutations in their variable regions before downregulating CXCR4 and cycling back to the LZ for further rounds of affinity-based selection (17–21). When FDCs are depleted, GC B cells and TFH cells die rapidly, whereas in the absence of CXCR4, GC B cells are confined to the LZ and have fewer somatic mutations (20, 22).

Several types of T cells populate the LZ and secrete a variety of cytokines that drive GC B cell differentiation and effector functions. The characteristics of TFH cells have recently been reviewed and will not be further addressed here (23–25). GC regulatory T cells have also been described (26). Foxp3^+^CD4^+^ T follicular regulatory cells (TFR) express CXCR5 and Bcl6 and act by downregulating CD80 and CD86 expression on B cells, thereby diminishing the strength of cognate B–T interactions (27, 28). Qa1-restricted ICOSL^+^CXCR5^+^ regulatory CD8 T cells interact directly with Qa1-expressing TFH cells (29). The balance between TFH and TFR sets the threshold at which B cells compete for T cell help; this in turn regulates B cell entry and dwell time in the DZ.

## Regulation of B Cell Selection in Germinal Centers

Most high-affinity naïve autoreactive B cells are anergized or deleted before the GC stage (30, 31). However, mechanisms need to be in place to purge those B cells that increase their autoreactivity or acquire autoreactivity *de novo* as a result of random somatic mutations occurring in the GC (31). How this is achieved is still not fully understood. Elegant studies have been performed in the hen egg lysozyme (HEL)/anti-HEL model using HEL variants with differing affinities and patterns of tissue expression. These studies have led to the paradigm that engagement of the BCR by self-antigen but in the absence of T cell costimulatory signals results in B cell death before the plasma cell stage. Tolerance can be broken if the self-antigen crossreacts with a foreign antigen, and B cells are therefore able to recruit help from anti-foreign T cells, or if the self-antigen is not present in high enough concentrations within the GC to mediate deletion (32).

Regulation of autoreactivity involving nucleic acid autoantigens is, however, more complex because low-affinity IgM anti-nuclear autoantibodies are required to opsonize and promote clearance of nucleic acid antigens that are shed from apoptotic cells; loss of this IgM can induce or accelerate autoimmunity (33). By contrast, IgG autoantibodies directed to nuclear autoantigens can penetrate tissues and initiate inflammatory cascades. There has been much work directed at understanding whether the class-switched autoreactive B cells that arise in systemic lupus erythematosus (SLE) are derived from naïve autoreactive B1, marginal zone, or follicular B cells that undergo clonal expansion either inside or outside the GC or whether they arise *de novo* by somatic mutation. Mice with site-directed transgenes that encode autoreactive immunoglobulin genes capable of class switching and somatic mutation have been used to address this question.

- D42 is an anti-dsDNA hybridoma derived from the NZB/W lupus-prone strain. Anti-DNA activity of D42 is conferred by its basic V_H_CDR3 region as well as by its associated light chain Vκ16–104. In non-autoimmune D42 heavy chain transgenic (D42hTg) mice, autoreactivity is regulated by clonal deletion at the immature stage, clonal anergy, and receptor editing. D42 hybridomas derived from these mice have low-affinity for DNA and use diverse light chains. In lupus-prone D42hTg NZB/W mice, clonal deletion initially appears intact but high-affinity IgG anti-DNA antibodies appear in the serum with age. In this strain, receptor editing of the light chain results in a preference in the naïve repertoire for Vκ4-55*01 that confers low-affinity polyreactivity. Nevertheless, nearly all IgG anti-DNA hybridomas from D42hTg NZB/W mice use Vκ16–104 rearranged to Jk5, a combination generated by receptor editing that confers high affinity for DNA. Thus, receptor editing can protect from autoimmunity but may also generate potentially dangerous antibodies (34–37). Using cell sorting and single cell analyses, we have shown that B cells expressing a restricted repertoire of light chains, including Vκk4–55*01, that confer no or low-affinity autoreactivity are positively selected into the naïve B cell pool of D42hTg NZB/W mice. By contrast, D42/Vκ16-104 expressing B cells are mostly deleted by the late transitional B cell stage, but are then preferentially selected and expanded in the GC as the mice age (38).
- The 3H9 heavy chain, also derived from an anti-DNA hybridoma, pairs with a wide variety of light chains to generate DNA and non-DNA binding, as well as low-affinity anti-cardiolipin antibodies (39). In non-autoimmune 3H9 heavy chain transgenic (3H9hTg) mice, autoreactivity is regulated by receptor editing and anergy (40). Those 3H9 B cells that do enter the GC and undergo somatic mutations fail to develop into plasma cells (41). By contrast, 3H9-encoded anti-dsDNA antibodies using pathogenic light chains arise in autoimmune strains (42). We showed in 3H9hTg NZB/W and NZW/BXSB mice that the GC and plasma cell repertoire is dominated by 3H9/Vκ5 pairs and that the Jκ region influences the avidity of such pairs for chromatin and the stringency for GC entry. The acquisition of somatic mutations then increases the affinity for chromatin and confers new binding for DNA or cardiolipin (43).

Taken together, the studies in D42 and 3H9 transgenic NZB/W mice show that failure to regulate GC entry and clonal expansion of rare naïve high-affinity autoreactive B cells (D42) and failure to regulate autoimmunity acquired by somatic mutation in the GC (3H9) can both contribute to loss of tolerance to nuclear autoantigens.

One approach to address the same question in humans is to express immunoglobulin genes from single B cells and test them for autoreactivity. Pioneering studies showed a high frequency of autoreactivity among early immature bone marrow B cells that is subsequently censored during the late immature and early transitional stages. These tolerance checkpoints are compromised in patients with SLE even during periods of remission (44, 45). Normal individuals also regulate autoreactivity within the IgM memory and bone marrow plasma cell compartments (46, 47). Sanz and colleagues showed that autoreactive VH4-34 encoded 9G4 idiotype^+^ B cells from normal tonsils have an anergic phenotype and fail to mature to a GC phenotype or enter the memory compartment. 9G4 B cells from SLE patients are overrepresented among GC and memory cells, suggesting tolerance failure in the GC (48). By contrast, single cell analyses have demonstrated that autoreactive B cells from the class-switched memory compartment can be derived from non-autoreactive precursors (49). Thus, in humans, as in mice, failure of regulation of autoreactive B cells can occur both at the GC entry checkpoint and at the post-somatic mutation checkpoint.

## Many Models of SLE are Characterized by Spontaneous GC Development

The Sle1 mouse that bears a region of chromosome 1 derived from the lupus-prone NZM2410 strain develops spontaneous GCs and high titers of anti-chromatin autoantibodies without systemic inflammation or clinical lupus (50, 51). The Sle1b locus harbors polymorphisms in genes from the signaling lymphocyte activation molecule (SLAM) family of receptors that contribute to loss of GC tolerance in a B cell-intrinsic manner. Paradoxically, Sle1b B cells manifest lower Ca2^+^ flux and less cell death than their wild-type counterparts, protecting them from negative selection in the GC (52). They also form poorer T–B conjugates, perhaps allowing them to interact more promiscuously with T cells (53).

Germinal center B cells upregulate the inhibitory FcRIIB receptor and this regulates the final differentiation of memory B and plasma cells. Failure to upregulate FcRIIB on GC B cells occurs in the Sle1 model (54) and in human lupus patients (55) resulting in loss of tolerance at the late B cell checkpoint. In addition, several lupus models have a polymorphism that confers lower levels of expression of FcRIIB (56); lupus in these strains is attenuated by restoration of FcRIIB on B cells (57).

Not surprisingly, TFH numbers influence GC reaction and regulate tolerance to autoantigens. One interesting model is deficient in Roquin1, an RNA-binding protein that post-transcriptionally represses expression of ICOS and the costimulatory molecule Ox-40 (58). Roquin1-deficient mice have increased numbers of TFH and develop GC-dependent autoimmunity with a lupus-like phenotype (59). In humans, increased expression of Ox-40L on myeloid antigen-presenting cells is induced by RNA-containing immune complexes and this in turn amplifies TFH responses once tolerance is broken (60). Failure of TFR can also induce lupus. For example, mice deficient in GC CD8 Tregs have large GCs, autoantibodies, and lupus-like glomerulonephritis (29).

Innate immune pathways also contribute to spontaneous GC formation in lupus mice. We showed that administration of a single dose of IFN-expressing adenovirus to young NZB/W and NZW/BXSB mice induces GC formation and autoantibodies in a T cell-dependent manner (61). Using single cell analysis and expression of 3H9 heavy/light chain pairs of interest, we further showed in NZW/BXSB mice that type-I IFN relaxes the stringency for GC entry and clonal expansion resulting in increased autoreactivity of the GC-derived repertoire (43).

Type-1 IFNs are induced through the activation of endosomal TLRs that specifically recognize nucleic acids. TLR7 is needed to generate antibodies against RNA antigens and cardiolipin, whereas TLR9 is needed for spontaneous generation of anti-dsDNA autoantibodies. TLR7-overexpressing mice develop severe inflammatory kidney disease and increased mortality (62), whereas disease is ameliorated in TLR7-deficient lupus mice (63). Surprisingly, TLR9-deficient lupus mice have increased autoimmune disease; this is associated with greater production of anti-RNA autoantibodies (63, 64). Lupus-prone mice deficient in both TLR7 and TLR9 or in their adaptor protein MyD88 do not develop either autoantibodies or lupus nephritis (63, 65).

Mice deficient in TLR7 on B cells have a significantly reduced GC response upon challenge with an RNA virus and have a relative decrease in the size of the DZ where proliferation and somatic mutation occur. This results in decreased production of high-affinity anti-viral antibodies (66). In lupus models, B cell-intrinsic TLR7 expression drives GC formation and autoantibody production (67). In the BWAS−/− model in which B cells are deficient in the Wiskott–Aldrich syndrome protein, GC formation depends on B cell-intrinsic TLR7 (64) but is unaffected by Type 1 IFN (American College of Rheumatology, Abstract 2870, 2014). In the pristane model of SLE, induction of TLR7 is downstream of Type-I IFN (68). These studies both suggest that the mechanism of action of TLR7 in GC formation and autoantibody induction is not mediated via induction of Type-I IFN. Using 3H9 NZW/BXSB male mice that bear two copies of TLR7, we showed that excess TLR7, like excess Type-I IFN, results in the failure of exclusion of high-affinity autoreactive B cells from the GC, with subsequent clonal expansion and somatic mutation of these cells that then can enter the plasma cell compartment (69).

## The Role of Baff in the Germinal Center

B cell-activating factor (BAFF) is made by stromal cells in the secondary lymphoid organs including FDCs. The interaction of BAFF with BAFF-R is required for B cell survival and regulates the selection of naïve autoreactive B cells whereas BAFF excess can induce lupus-like autoimmunity (70). The GC is in general a BAFF poor environment, and expression of BAFF receptors is also downregulated, with BAFF-R being the sole receptor on GC B cells (71). BAFF and BAFF-R KO mice form smaller GCs that decline prematurely, indicating that BAFF is needed for GC maintenance (72). The mechanism for this dependence is not yet clear. BAFF influences ICOSL expression on B cells, thus regulating the ability of B cells to promote TFH expansion during GC development (73). TFH, in turn, produce BAFF and this may enhance the survival and selection of high-affinity B cells (74). Acting via a receptor other than BAFF-R, BAFF is required for the development of a mature FDC reticulum (72). Furthermore, activated T cells are also responsive to BAFF, releasing IFNγ that amplifies autoimmune effector responses (75). TFH cells express both BAFF-R and BCMA. In a lupus-prone strain with T cell-specific BCMA deficiency, TFH cells are expanded and signaling through BAFF-R results in enhanced IFNγ release (76). Whether BAFF modulates the selection of autoreactive B cells in the GC either in mice or humans is not yet known.

## Extrafollicular Clonal Expansion and Somatic Mutation in SLE

Extrafollicular B cells can undergo clonal expansion and limited somatic mutation. Although T cell help is usually rather limited in EF foci, TFH-like cells have been found in these foci in several lupus-prone mouse strains, raising the possibility that somatic mutations leading to high-affinity autoreactivity can occur in these sites without the usual regulatory mechanisms in place (77, 78). In NZW/BXSB mice, GCs are found early in the disease but then disappear around the time of disease onset and are replaced by a disorganized splenic architecture with many EF TFH cells (79). Induction of class-switched autoreactivity outside GCs also occurs in BAFF transgenic mice; this requires MyD88 but not T cell help suggesting that under some circumstances, autoreactivity can be driven solely by innate mechanisms (80). Whether humans with lupus generate autoantibodies through this mechanism has not yet been determined. Recent studies in the 9G4 system have shown that during SLE flares, activated naïve B cells are the source of oligoclonal expansions of plasma cells with only low frequencies of somatic mutation, suggesting that some autoantibodies may derive from EF sources in humans (81).

## Cell Death in Germinal Centers

Germinal center B cell survival is a result of the balance of pro-survival and pro-apoptotic molecules whose expression is regulated by affinity-based BCR signals, extrinsic signals from other cells, including cytokines and contact-dependent signals, and stochastic effects, that are either random or reflect local signals at critical times (11). The death molecule Fas is highly upregulated in GC B cells and its loss increases the size of the GC but decreases affinity maturation as low-affinity antigen-specific B cells fail to die (82). Similarly, transgenic overexpression of the anti-apoptotic Bcl2 protein results in survival of low-affinity antigen-specific B cells and B cells cross-reactive to self-antigens (83). More recent studies have dissected the role of individual Bcl2 family members in GC and post-GC B cell survival and selection (84).

Given the high death rate in the GC, specialized mechanisms are in place to clear dead cells before necrotic material activates innate pathways that could induce autoimmunity. Apoptotic cells are opsonized by soluble molecules, such as IgM, C1q, and MFGE8, and/or are rapidly cleared by highly phagocytic tingible body macrophages that express a large variety of scavenger receptors (85). C1q deficiency is a highly penetrant genetic cause of SLE in humans (86). MFGE8 recognizes phosphatidyl serine on apoptotic cells in the GC. Its absence in mice results in the induction of autoantibodies with age; this loss of tolerance is accelerated by repeated immunizations that induce GC formation (87).

## Targeting the GC in SLE

Considering the important role of the GC in the acquisition of autoreactivity in SLE, targeting of the GC has been a major focus of therapeutic endeavor. Early studies using the costimulatory antagonists, anti-CD40L, to abort ongoing GCs or CTLA4Ig to prevent GC formation were highly effective at preventing lupus onset in mouse models in which GCs predominate as a source of autoantibodies. A short-term combination of CTLA4Ig and anti-CD40L was even more effective than either agent alone (88). We showed that in NZB/W mice, this was due to tolerance induction in the GCs, preventing the formation of cross-reactive autoantibodies upon immunization with foreign antigen (89). Similar restoration of tolerance occurs after administration of an agonist antibody to CD137 (4-1BB) (90). Other GC targets include TLR7 and TLR pathways, BAFF, ICOS that is important not only for GC development but also for the function of effector T cells, and IL-21 that is expressed both by GC and EF TFH cells. An alternate strategy is to increase clearance of nucleic acids using RNAse or DNAse.

In mouse models, therapies targeting the GC lose efficacy once long-lived autoreactive memory and plasma cells have already developed (88). Class-switched memory cells do not need to reenter the GC to rapidly differentiate into plasma cells, and long-lived plasma cells require neither antigen nor T cells to survive. GC-directed therapy might therefore be most effective in the early stages of disease or as maintenance therapy during disease quiescence. Another potential use of GC-directed therapy might be to prevent the emergence of autoantibodies that arise in ectopic tissue GCs during inflammation and are directed to tissue antigens, such as vimentin (91). Finally, it is possible that targeting ectopic TFH could prevent ongoing formation of EF foci of short-lived plasma cells.

## Conclusion

Defective tolerance induction in the GC is a feature of many SLE models and can be due both to impaired selection of naïve autoreactive B cells into the GC and impaired regulation of B cells that acquire autoreactivity by somatic mutation. Defects in GC T cells and insufficient clearance of the large numbers of dead cells that are generated in the GC can lead to loss of tolerance to nuclear autoantigens. Impaired signaling through the BCR leading to insufficient B cell death and excess innate signals leading to a decrease in stringency of B cell selection both lead to increased B cell autoreactivity. Many strategies are currently being tested for targeting of the GC, but the clinical efficacy and optimal applications of this approach remain to be tested in humans.

## Conflict of Interest Statement

The authors declare that the research was conducted in the absence of any commercial or financial relationships that could be construed as a potential conflict of interest.
